# 

**Published:** 1919-04

**Authors:** 


### ANNOUNCEMENTS

*Texas State Dental Society* will meet in Waco, April 22-25, 1919.

*Illinois State Dental Society* meets in Peoria, May 13th to 16th inclusive.

*Indiana State Dental Society* meets in Indianapolis, May 20th to 23d inclusive.

*Northern Ohio Dental Society* meets June 2d to 4th, at Hotel Statler in Cleveland.

*Kentucky State Dental Association* meets June 9th to 12th inclusive, in Louisville. This is the fiftieth anniversary meeting and an unusual meeting is in preparation.

*New York State Dental Society* will meet June 12th to 14th, in Syracuse, at the Hotel Onondaga.

---

### AMERICAN INSTITUTE OF DENTAL TEACHERS

The annual meeting of the American Institute of Dental Teachers was held at the Piedmont Hotel, Atlanta, Georgia, January 28-30. The following officers were elected for the ensuing year: President, Dr. R. W. Bunting, Ann Arbor, Mich.; Vice-President, Dr. Arthur D. Black, Chicago, Ill.; Secretary-Treasurer, Dr. Abram Hoffman, Buffalo, N. Y.; Executive Board, Dr. G. S. Millberry, San Francisco; Dr. A. H. Hipple, Omaha; Dr. A. E. Webster, Toronto. The next regular meeting will be held at Detroit, Mich.—ABRAM HOFFMAN, *Secretary*, 381 Linwood Avenue, Buffalo.

---

### MODERN DENTISTRY FOR THE LAITY.

This is a well-printed volume of 136 pages by Alfred Armstrong Crocker, containing a most creditable exposition of the subject of Industrial Dentistry as it has been worked out by various business concerns for the benefit of their employes. The articles are very well written and present the subject with remarkable clearness and unusual insight of the professional bearings of the subject. It is pubished by the DENTAL REGISTER and may be had of the Sam'l A. Crocker Co., 20 W. 7th St., Cincinnati, Ohio.

---

THE UNITED STATES—A GOING CONCERN.

There is a passing uneasiness in the public mind in the United States which is manifesting itself in various ways. The war has shaken individuals out of their former easy habits of thinking—or not thinking—and the acquisition of knowledge is proving acutely painful to many kinds of brains. Some of them are unequal to the strain of absorbing truth. They are turning sour, pessimistic or bolshevist, according to the temperament of the individual. One type of mind that seems to be increasing in numbers is that which would throw away all the good that has been acquired through a century of effort, to embrace notions that can not stand ten minutes of quiet analysis. This kind of hair-trigger American is reveling now in the chaotic state of affairs, hoping to see a complete overturn in which he will stand a chance of grabbing some advantage which he can not hope to acquire in honest competition with his fellows.

The fault, however, does not lie with the shallow-pated or the merely unripe intellects. Some of the strongest business men in the country are behaving as though their brains had deserted them. Instead of concentrating upon the new problems that are presented and quietly conferring upon ways and means to co-operate on a sensible course of action, they are throwing fuel upon the bolshevik bonfires by ill-considered speech and unwise acts. Others have drawn into their shells, refusing to loosen their pocketbooks for the sake of keeping the wheels turning. They excuse this turtle-like maneuver by asserting that "no one knows what is coming," and "we must wait to see what the peace conference will do," or similar nonsense.

Of course, no one knows what is coming. But is it not presumable that the richest, strongest, most solvent, most active and most independently-placed nation in the world will continue on the map? Surely that is not a violent assumption, although, strictly speaking, it is no more capable of demonstration than any other future proposition. It is sufficient, however, for the ordinary American if he will but stop to think, that he and his fate are bound up in the fate of the country. If the United States is to go down, the average American would just as soon go down with it; and if the United States is to go forward, the average citizen is satisfied to go along, rather than seek his fortune in other countries.

The banks are bulging with money. The people of this country are rich. Their government borrowed billions, but it borrowed from the people who have recovered most of the money in business and at the same time hold the bonds, which are drawing interest. The bankers of the country are cautious—a good trait in bankers, if it does not degenerate into mere sheepish timidity. Would it not be well for the bankers to examine themselves and the country and ask whether they are not a bit too cautious for the best interest of all concerned? Does the United States depend upon the peace conference after all? Is it not rather a fact that the United States has been held back too long in its normal domestic development until it is too cramped? The 100,000,000 individuals of this country want more railroads, more buildings, more homes, more schools, more churches, more suburbs, more farms, more gardens, more of everything that brings comfort. Why shouldn't the people have what they want when they have the money to buy it?

With all due respect to the strength of the late German empire, it seems to us that many Americans are conceding too much to the prowess of the enemy when they are afraid to go forward in business because of "unsettled conditions." Are they afraid that Germany will come back and start another war? Not a chance! Nor will anybody else start a war that need bother the United States. This mighty, eager nation is suffering now from nothing more than a false state of mind on the part of its own people. They have been shaken and are uneasy. Let them think twice and they will perceive that the old United States is in fact, bigger and richer than ever, with greater prestige abroad than ever before. The victory of liberty did not hurt the United States, however terribly it wounded Germany. The free nations will give us a better market than Germany ever dreamed of giving. All that Americans need to do now, if they wish prosperity, is to cut out foreign notions of government, throw away their worry over the peace conference and the settlement of foreign questions, roll up their sleeves and get busy.—*Washington Post*, January 29, 1919.

